# Polyelectrolyte Cylindrical Brushes in Hairy Gels

**DOI:** 10.3390/polym15153261

**Published:** 2023-07-31

**Authors:** Ekaterina B. Zhulina, Oleg V. Borisov

**Affiliations:** 1 Institute of Macromolecular Compounds of the Russian Academy of Sciences, 199004 St. Petersburg, Russia; 2 Institut des Sciences Analytiques et de Physico-Chimie pour l’Environnement et les Matériaux, UMR 5254 CNRS UPPA, 64053 Pau, France

**Keywords:** polyelectrolyte gels, molecular brushes, hairy gels

## Abstract

We considered dispersions of cylindrical polyelectrolyte (PE) brushes with stiff backbones, and polymer-decorated nanorods with tunable solubility of the brush-forming PE chains that affected thermodynamic stability of the dispersions. We focused on thermo-induced and deionization-induced conformational transition that provokes loss of aggregative dispersion stability of nanorods decorated with weakly ionized polyions. A comparison between theoretical predictions and experiments enabled rationalization and semi-quantitative interpretation of the experimental results.

## 1. Introduction

Molecular brushes, among other types of branched macromolecules, have been extensively studied both theoretically and experimentally with the aim of designing and fabricating advanced functional nanostructured materials, elastomers and gels with outstanding mechanical properties [1,2,3,4,5,6,7,8,9,10,11].

In contrast to linear macromolecules, strong intra-molecular interactions between densely grafted side chains specify the properties of molecular brushes in solutions and in the melt state. Analytical theory [12], supported by self-consistent field [13,14] and coarse-grained Brownian dynamics and Monte Carlo computer simulations [15,16,17,18,19,20,21,22] have enabled rationalizing the relationships between branched macromolecular architecture and properties of materials, including bulk elastomers and hairy gels. The latter can be obtained by physical or chemical cross-linking of bottlebrush molecules or by tuning solubility of individual chains in bottlebrushes leading to their association into networks due to loss of the solution thermodynamic and aggregative stability.

In contrast to conventional gels with linear (undecorated) strands, hairy gels composed of physically or chemically cross-linked bottlebrushes [23,24,25] exhibit two distinct regimes of behavior: (i) a hollow mesh regime in which the side chains from neighboring strands do not considerably overlap, and solvent in the mesh is distributed unevenly, and (ii) a filled mesh regime in which side chains overlap and form semi-dilute solution with an almost uniform solvent distribution. The hairy gels with extended backbones are predicted to attain maximal swelling ratios at the boundary between hollow and filled mesh regimes [26]. Ionization of the side chains would lead to extra stretching of the backbones compared to neutral counterparts, and the corresponding modification of the hairy gel behavior in both regimes. Strong intermolecular (Coulomb) repulsions between charged side chains could stretch the backbone of the bottlebrush up to its contour length, transforming it locally (and globally) in a cylinder. A PE cylindrical brush could therefore serve as a model of hairy gel strands in low salt conditions under which the electrostatic repulsions between the side chains could stretch the strand backbone up to its contour length, and concomitant significant increase in the gel swelling coefficient compared to neutral counterparts. However, a vast majority of the theoretical studies on bottlebrushes deal with neutral systems, while computational studies of molecular brushes with ionized grafts are relatively sparse [27,28,29,30].

Another strategy to form hairy gels is to vary the temperature in a solution of molecular brushes that would lead to the collapse of the side chains and trigger the hydrophobic attraction between bottlebrush molecules. In the case of PE bottlebrushes with rigid backbones and polymer-decorated cylindrical nanorods (e.g., carbon nanotubes [31,32], cellulose nanocrystals [33,34], gold cylindrical nanoparticles [35,36], etc.), the necessary (but not sufficient) condition for the solution gelation is, therefore, the onset of the collapse of the side chains. The latter depends on the structure of the polymer layer and its thickness.

The original scaling models of PE brushes [37,38,39,40,41] predicted three major brush regimes in all geometries: osmotic (at low salt concentration), salt-dominated (at intermediate salt concentrations), and quasi-planar (at large amounts of salt with a predominance of non-electrostatic monomer–monomer interactions) with a narrow charged regime [37] separating individual tethered polyions from osmotic brushes [40]. The scaling theory provided power law dependences for the brush thickness and polymer density profile as functions of the molecular parameters: degree of ionization and molecular weight of the tethered polyions, their grafting density, the radius of the grafting surface, and solvent strength.

Further extensions/modifications in the PE brush theory included: shear and lubrication, pH- and salt responsiveness, the effect of the molecular parameters (e.g., the polydispersity of polyions, their rigidity, charge distribution, and architecture), size and valence of counterions and salt ions, solvent structure and its thermodynamic quality, etc. Leaving an enormous number of PE brush studies to reviews (see, e.g., refs. [42,43]), we mention here only few publications on scaling models [44,45,46,47], analytical [48,49,50] and numerical [51,52] Poisson–Boltzmann frameworks, and computer simulations [20,53,54,55,56,57] that have illuminated important aspects of the PE brush behavior.

In this study, we focus on PE bottlebrushes with rigid backbones that give rise to hairy gels in two possible ways: (i) cross-linking of swollen bottlebrushes, and (ii) onset of collapse of side chains in solutions of bottlebrush molecules. In the former system, the hairy gel is isotropic and has a well-defined mesh size, while in the latter system, gelation can lead to mesh polydispersity and orientation of the strand backbones. Moreover, in the case of polymer-decorated cylindrical nanoparticles (NPs), the surface of NP could bear ionizable groups that affect the onset of the collapse of the PE brush layer.

The rest of the paper is organized as follows: In Section 2, we start by formulating the model of a cylindrical PE brush with a rigid backbone, briefly review the scaling results for isolated PE brush in salt-free and salt-added aqueous solutions. Further, we consider the collapse of the cylindrical PE brush provoked by a decrease in the solvent quality for the brush-forming chains and assisted by their electrostatically driven adsorption on the oppositely charged backbone. At the end of this section, we contrast theoretical predictions against selected experimental results. In Section 3, we formulate our conclusions.

## 2. Results and Discussion

### 2.1. Model of the PE Cylindrical Brush

**Figure 1:**
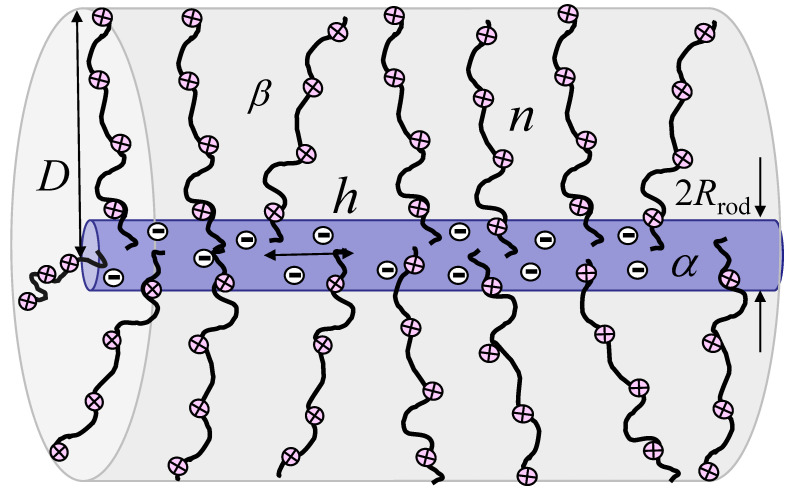
Schematic of cylindrical polycationic brush with the polymerization degree *n* of tethered polyions and degree of ionization β (i.e., with partial charge eβ per monomer unit), and linear distance *h* between neighboring grafts.

We consider a cylindrical PE brush consisting of a rigid backbone (a cylindrical rod or radius $R_{rod}$) with multiple flexible side chains, each with the degree of polymerization *n*, tethered to the backbone at axial distance *h* between neighboring grafts (Figure 1). The monomer unit size *a* in grafted chains is on the order of the Kuhn segment length, and the fraction 0≤β≤1 of the monomer units carry elementary positive charges *e*. The Bjerrum length $l_{B}=e^{2}/\left(\varepsilon k_{B}T\right)$ is assumed to be on the order of *a* (i.e., for $l_{B}\approx 0.7$ nm in aqueous solution with dielectric permeability ε≃80, ratio $l_{B}/a≃1$). The backbone can be charged (oppositely or similarly to the tethered polyions) with surface number charge density α≥0. The solution contains monovalent salt ions with concentration $c_{s}$ which specifies the Debye screening length as $r_{D}={\left(8\pi l_{B}c_{s}\right)}^{-1/2}$.

If the backbone is neutral (α=0) and polyions are very weakly charged, then the side chains acquire Gaussian coil conformation with the size $R_{G}≅an^{1/2}$ but get stretched with respect to their unperturbed (Gaussian) dimensions due to short-range inter-molecular repulsive interactions beyond the overlap threshold, $h\leq R_{G}$. If side chains are charged up to $\beta \geq {\left(l_{B}/a\right)}^{1/2}n^{-3/4}$, then at low grafting density they behave as isolated polyions stretched by intra-molecular Coulomb repulsions up to the size $R_{e}≅\beta^{2/3}n{\left(l_{B}/a\right)}^{1/3}≃\beta^{2/3}n$. Each polyion can be envisioned as a string of electrostatic blobs of size

(1) $$\xi_{e}≃a\beta^{-2/3},$$

each blob comprises $g_{e}≅{\left(\xi_{e}/a\right)}^{2}$ monomer units, i.e., the chain segment inside the blob is not perturbed by Coulomb repulsions and keeps its Gaussian size, the energy of intramolecular Coulomb repulsions is on the order of ∼$k_{B}T$ per blob. As the grafting density increases, at $h\geq R_{e}$ the inter-molecular Coulomb repulsions first induces orientation of individual polyions in the direction perpendicular to the backbone (similar orientation was previously predicted for planar polyelectrolyte brushes in ref. [40]). Upon further increase in grafting density, at $h\leq R_{e}$ the inter-molecular Coulomb repulsions dominate over the intra-molecular ones and cause additional stretching of the tethered polyions in the radial directions beyond the size $R_{e}$. Hence, the condition

(2) $$h\leq a\left\{\begin{array}{cc} \beta^{2/3}n{\left(l_{B}/a\right)}^{1/3}≃\beta^{2/3}n & \;\mathrm{regime}\;\mathrm{IS}\\ n^{1/2} & \;\mathrm{regime}\;G \end{array}\right.$$

specifies the onset of the brush regime, where the inter-molecular interactions dominate over the intra-molecular ones and cause extra stretching of the tethered chains. The first and second lines in Equation (2) correspond to $\beta >n^{-3/4}$ or $\beta <n^{-3/4}$, respectively, and notation IS indicates “isolated stretched” polyion.

If surface backbone charge density α≠0, then the properties of the PE brush would be modified, as discussed below.

### 2.2. PE Cylindrical Brush with Uncharged Backbone in Salt-Free Solution

The equilibrium properties of a salt-free cylindrical PE brush have been discussed in a number of publications, and we only summarize here the relevant results.

**Figure 2:**
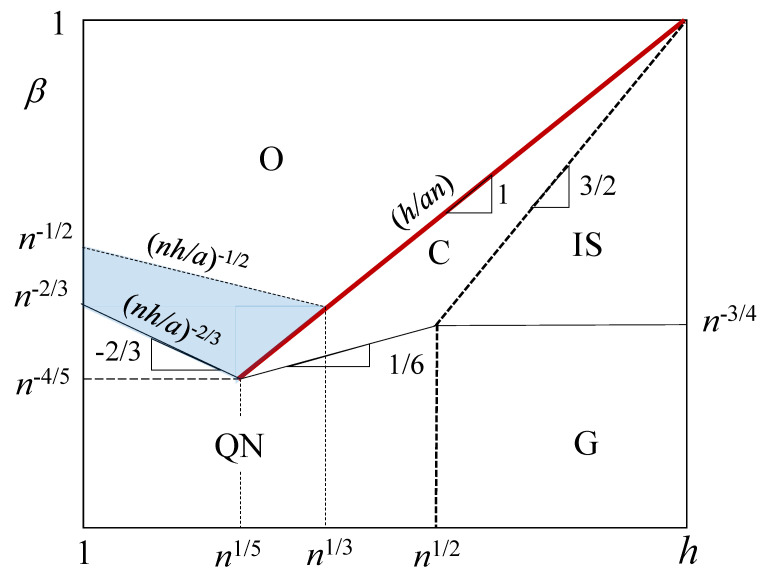
Scaling-type diagram of states for cylindrical PE brush in salt-free solution in (β,h) log–log coordinates, $l_{B}=a$, and α=0 (uncharged backbone). Red line β≅h/na corresponds to the onset of counterion condensation in the brush volume. Overlap thresholds for side chains, $h=R_{e}$, shown by dashed lines, separate regimes of individual polyions (IS and G) from brush regimes (O,C,QN). Theta-solvent conditions with respect to short-range van der Waals monomer–monomer interactions.

In Figure 2 we reproduce the scaling-type diagram of states for cylindrical PE brush under theta-solvent conditions and uncharged backbone (α=0) in β, *h* log–log coordinates. To the right of the boundary $h≃R_{e}$ marked by the dashed lines (Equation (2)), a cylindrical brush with a stiff backbone has nonoverlapping side chains with the average end-to-end distance $D≃R_{e}$ (regime IS if $h>R_{e}$ or regime G with $D≃R_{G}≃an^{1/2}$). To the left of the dashed lines and relatively large *h*, the brush is found either in charged (C) or quasi-neutral (QN) regimes. Notably, in the charged regime C distance h/βn remains larger than $l_{B}$. A narrow intermediate regime C separates isolated polyions (IS) from the osmotic brush (O), which is the major regime of a salt-free PE brush. In Table 1 we present thickness *D* and polymer density profile c(r) in various regimes of the diagram [58].

In scaling terms, the onset of counterion condensation ($h/\beta n≃l_{B}$) occurs at the boundary between charged (C) and osmotic (O) regimes, in which the dominant part of counterions condense inside the brush volume. Specifics of counterion distribution upon crossing the C-O boundary are beyond the scaling model adopted in this paper. (More details about counterion distribution near charged objects can be found in e.g., ref. [59]). However, a crossover of the brush thickness *D* at the C-O boundary suggests that the applied scaling model correctly accounts for the major brush rearrangement associated with counterion condensation.

Notably, in the charged regime C, the polymer density profile in cylindrical PE brush is not described by a single power law dependence [60]. Due to the intermediate nature of the charged regime C, we consider below mostly the osmotic regime O, the main regime of a salt-free PE brush.

In the following, we focus on the lower part of the osmotic regime O (shaded blue in Figure 2), for which thickness *D* of cylindrical PE brush demonstrates a single dependence in the whole salt-dominated regime, $D\left(c_{s}\right)∼c_{s}^{-1/4}$. In the upper part of osmotic regime O (above the blue area), the $D(c_{s})$ dependence could demonstrate multiple exponents due to the separation of the intra- and intermolecular repulsions in tethered polyions.

### 2.3. PE Cylindrical Brush with Uncharged Backbone in Salt-Added Solution

In the cylindrical geometry, area per chain s(r) at distance *r* from the backbone increases as s(r)≃hr. In the salt-added solution, tethered polyions are exposed to the differential osmotic pressure of mobile ions,

(3) $$\frac{∆\Pi \left(r\right)a^{3}}{k_{B}T}=c_{+}\left(r\right)+c_{-}\left(r\right)-2c_{s}=2c_{s}\left(\sqrt{{\left(\frac{\beta c(r)}{2c_{s}}\right)}^{2}+1}-1\right)$$

Here

(4) $$c_{\pm }\left(r\right)=c_{s}\left[\pm \frac{\beta c(r)}{2c_{s}}+\sqrt{{\left(\frac{\beta c(r)}{2c_{s}}\right)}^{2}+1}\right]$$

are concentrations of mobile counterions and coions at distance *r* from the backbone, βc(r) is concentration of immobile charges on polyions, and Equation (3) presumes PE brush local electroneutrality, $c_{+}\left(r\right)\approx c_{-}\left(r\right)+\beta c\left(r\right)$.

The electrostatic stretching force

(5) $$\frac{f_{⊥}\left(r\right)}{k_{B}T}≃s\left(r\right)∆\Pi \left(r\right)≃hr∆\Pi \left(r\right)$$

balanced with the elasticity of polyions determines local tension t(r) in polyions, polymer density profile c(r), and the brush thickness *D*.

**Figure 3:**
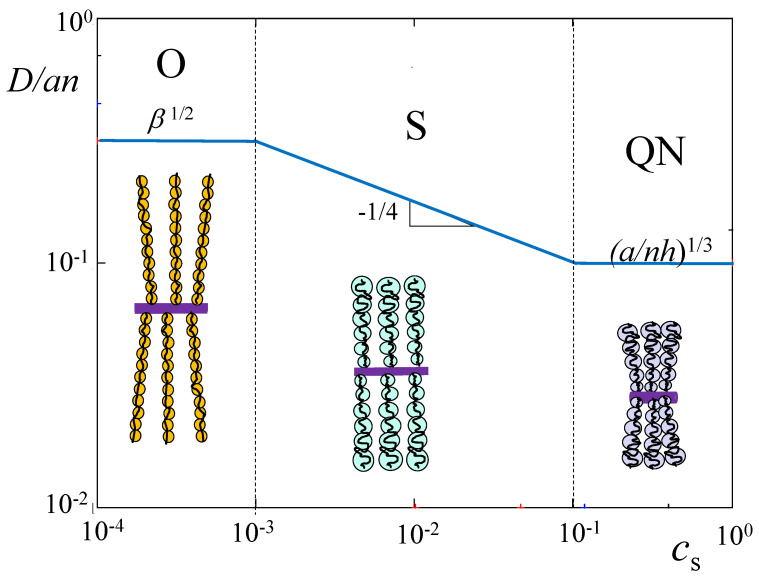
Reduced thickness D/an of a cylindrical PE brush as function of salt concentration $c_{s}$ for fixed $nh/a=10^{3}$, and β=0.1 (blue region in Figure 2), α=0.

A mean-field picture of the salt-dominated cylindrical brush [58] implies that charges on polyions and small mobile ions are uniformly spread throughout the brush to ensure the brush’s local electroneutrality. This approximation leads to a single power law dependence for the brush thickness *D* as a function of salt concentration $c_{s}$, $D∼{\left(\beta^{2}/c_{s}\right)}^{1/4}$, which is obtained by balancing the Gaussian elasticity of polyions with a differential osmotic pressure of salt ions. This classic picture holds if the degree of ionization β is relatively low (blue area in the diagram of states in Figure 2), and in this case the PE brush demonstrates only three regimes upon additions of salt ions: osmotic O, salt-dominated S, and quasi-neutral QN (see Figure 3). In Table 2, the equilibrium parameters of PE cylindrical brush are collected in these three regimes.

### 2.4. Globular State of the Cylindrical PE Brush

Above, we briefly reviewed the scaling model of PE cylindrical brush with intent to apply the results to hairy gels designed by either cross-linking of stiff backbones of molecular brushes or temperature-induced condensation in a solution of such macromolecules. In the latter case, the starting point for gelation is the onset of the temperature-induced collapse of the cylindrical PE brush.

Inferior solubility of polyions leads to the brush conformational re-arrangements and eventual collapse of the polymer layer. It is clear that an increase in hydrophobicity of the polymer chains due to inferior solvent strength would lead to smaller values of salt concentration $c_{s}$ at which the layer collapses. Therefore, the transition into a collapsed state of the polymer layer is governed by both, deviation τ=(θ−T)/T>0 from θ-temperature, and salt concentration $c_{s}$. Moreover, it also depends on the molecular parameters: degree of the polyion ionization β, DP *n* of tethered macromolecules, and linear distance *h* between grafts. Different scenarios of collapse at arbitrary values of β are out of the scope of this study, and we limit ourselves to relatively small values of β (blue area in the diagram of states in Figure 2). In this range of parameters, brush thickness $D>R_{e}$ at any salt concentrations $c_{s}$, and $D(c_{s})$ dependence demonstrates only three regimes upon increasing $c_{s}$: osmotic (O), classic salt-dominated (S), and quasi-neutral (QN), with $D(c_{s})$ depicted in Figure 3.

A cylindrical PE brush in the globular state has a uniform volume fraction *c* of monomer units, the concentration correlation length ξ≃a/c, and thickness $D_{\mathrm{collapsed}\;}≃$ ${\left(na^{3}/hc\right)}^{1/2}$. Losses in the free energy associated with the external brush boundary gives rise to the hydrophobic attraction between polymer-decorated nanorods and their associations. With accuracy of the logarithmic prefactors, polyions have the elastic free energy $F_{elalstic}/k_{B}T≃D_{\mathrm{collapsed}}^{2}/a^{2}n$ ≃a/hc per chain, which is negligible if c>a/h. The equilibrium value of *c* is specified by the condition

(6) $$\Pi \approx \Pi_{ion}+\Pi_{\mathrm{volume}}=0$$

with ion contribution $\Pi_{ion}=∆\Pi$ (Equation (6)), and $\Pi_{\mathrm{volume}}a^{3}/k_{B}T$≃$-\tau c^{2}+c^{3}$ due to the attractive binary (with second virial coefficient $-a^{3}\tau <0$, first term) and repulsive ternary (with third virial coefficient $≃a^{6}$, second term) monomer–monomer contacts.

In the box-like model of osmotic brush (with c(r)=const and predominance of own counterions over added salt ions), $\Pi_{ion}a^{3}≃k_{B}T\beta c$, and Equation (6) reduces to

$$\beta -\tau c+c^{2}=0$$

with the omitted numerical coefficients on the order of unity. Solution of this equation, $c≃\tau (0.5+0.5\sqrt{1-4\beta /\tau^{2}})$, indicates that in a stable globule with $\tau \gg \beta^{1/2}$, the equilibrium concentration c≈τ. As τ decreases and approaches $\beta^{1/2}$, the globular state loses stability, and the PE brush thickness exhibits transition from $D_{\mathrm{collapsed}\;}$ to $D≃an\beta^{1/2\;}$ (regime O, in which PE brush is stretched by the osmotic pressure of counterions). The value of $\tau ≃\beta^{1/2}$ can be thereby associated with the onset of temperature-induced instability of PE brush in the osmotic regime (O),

(7) $$\tau_{\mathrm{collapse}}^{\left(O\right)}≃\beta^{1/2}$$

Notably, a similar power law dependence, $\tau_{\mathrm{collapse}}≃\beta^{1/2}$, is obtained by equating size $\xi_{\mathrm{osm}}$$≃a\beta^{-1/2\;}$ of the elastic blob in regime O to size $\xi_{T\;}≃a/\tau$ of the thermal blob.

**Figure 4:**
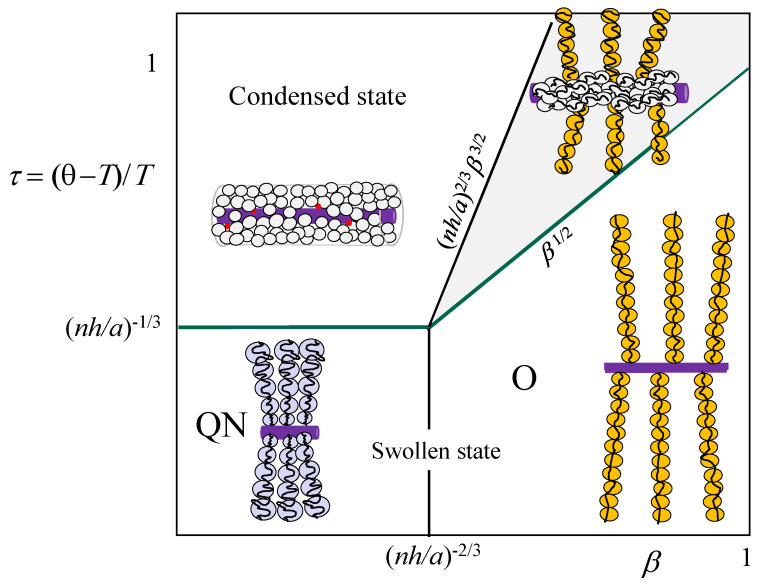
Scaling-type diagram of states for osmotic cylindrical PE brush in salt-free solution in (β,τ) log–log coordinates, $l_{B}=a$, α=0, with two phase region constructed after ref. [44]. Below green lines PE brush is in a swollen state. Above green lines cylindrical PE brush is collapsed, with collapse starting from outside of quasi-neutral brush and via two-phase region (shaded grey) for osmotic brush.

Account of polymer density decay c(r)$∼r^{-1}$ in the osmotic PE brush leads to a sharp but gradual transition between the osmotic and collapsed states via an intermediate two-phase region [44]. In Figure 4 we present the scaling-type diagram of states of osmotic (salt-free) brush in τ=(θ−T)/T, β log–log coordinates, the two-phase region is shaded grey. Insets illustrate the blob structure of tethered polyions.

In the salt-dominated regime S and quasi-neutral regime QN, $\Pi_{ion}a^{3}≃k_{B}T\beta c^{2}/c_{s}$, and Equation (6) reduces to

$$\left(\beta^{2}/c_{s}-\tau \right)c+c^{2}=0$$

to specify the average volume fraction *c* of monomer units in globular state as $c≃(\tau -\beta^{2}/c_{s})$.

In the brush quasi-neutral regime QN ($c_{s}>$$\beta^{2}{\left(hn/a\right)}^{1/3}$), nonelectrostatic interactions (i.e., ternary monomer–monomer contacts with the third virial coefficient $≃a^{6}$) dominate over the electrostatic repulsions between charges. In this scenario, the temperature-mediated size of the thermal blob, $\xi_{T}≃a/\tau$, becomes equal to the size of the last elastic blob, $\xi \left(D\right)≃{\left(a^{2}nh\right)}^{1/3}$, when $\tau ≃{\left(a/nh\right)}^{1/3}\gg \beta^{2}/c_{s}$, and thereby

(8) $$\tau_{\mathrm{collapse}}^{\left(\mathrm{QN}\right)}≃{\left(a/nh\right)}^{1/3}$$

is assimilated with the onset of the brush collapsed state. Further, increase in $\tau >\tau_{\mathrm{collapse}}^{\mathrm{QN}}$ leads to gradual propagation of the collapsed state down inside the brush, and the corresponding decrease in the brush thickness to

(9) $$D_{\mathrm{collapsed}}≃{\left(na^{3}/h\tau \right)}^{1/2}$$

In the salt-dominated regime S, the collapse of the polymer layer is associated with temperature-induced variations in the second virial coefficient $va^{3}≃\left(-\tau +\beta^{2}/c_{s}\right)$ of monomer–monomer interactions. A decrease in $\tau ≳\beta^{2}/c_{s}$ leads to onset of collapse with

(10) $$\tau_{\mathrm{collapse}}^{\left(S\right)}≳\beta^{2}/c_{s}+{\left(a/nh\right)}^{1/3}≃\beta^{2}/c_{s}$$

Lack of crossover in $\tau_{\mathrm{collapse}}$ at the boundary between osmotic (O) and salt-dominated (S) regimes suggest that the two-phase the region extends in the salt-dominated regime. Extension of the transition temperature $\tau_{\mathrm{collapse}}^{\left(O\right)}$$≃\beta^{1/2}$ to regime S provides a smooth crossover between transition temperatures, $\beta^{1/2}$ ≃ $\beta^{2}/c_{s}$ at $c_{s}≃\beta^{3/2}$. We emphasize that values of $\tau_{\mathrm{collapse}}$ are estimated with the accuracy of numerical coefficients (and nonpower law dependences) to highlight the general trends in PE brush behavior.

**Figure 5:**
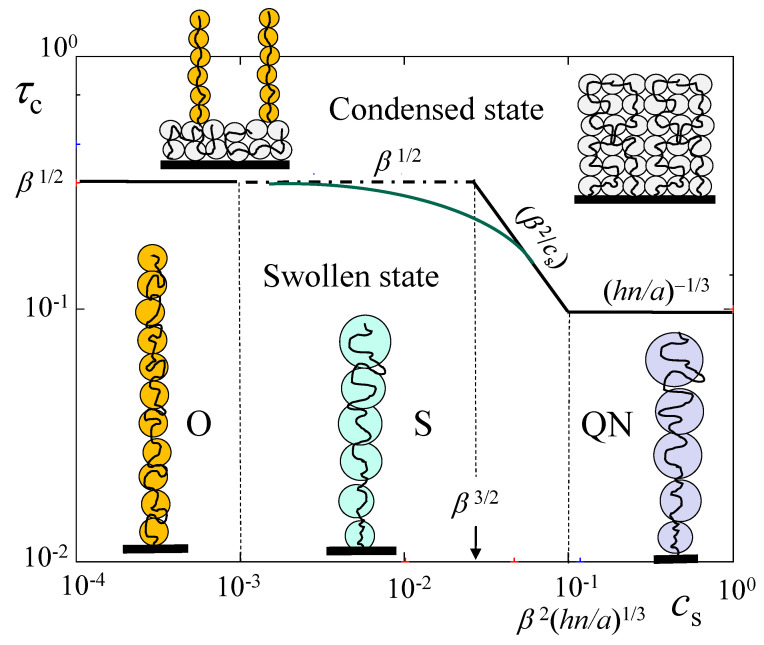
Power law dependences for onset of collapse transition reduced temperature, $\tau_{collapse}\left(c_{s}\right)$, for PE cylindrical brush with degree of ionization β=0.1, $\alpha =0,nh/a=10^{3}$ (blue area in Figure 2). Brush thickness $D(c_{s})$ in swollen state is indicated in Figure 3. Schematics of blob structure in tethered polyions are indicated in regimes O (in light brown), S (green) and NQ (violet), and collapsed state (grey). Lack of crossover in $\tau_{collapse}\left(c_{s}\right)$ at the boundary between regimes O and S is indicated by dash-dotted line, extending onset of collapse, $\tau_{collapse}≅\beta^{1/2}$, beyond regime O.

In Figure 5, we illustrate the onset of the PE brush collapse, $\tau_{\mathrm{collapse}}$, as a function of salt concentration $c_{s}$ in log–log coordinates for weakly charged polyions (blue area in the diagram of states in Figure 2). The equations for $\tau_{\mathrm{collapse}}$ are presented above the transition lines. Dash-dotted line indicates extension of $\tau_{\mathrm{collapse}}^{(O)}$$≃\beta^{1/2\;}$ into the salt-dominated regime S. At $\tau <\tau_{\mathrm{collapse}}$ (below the transition lines) the PE brush is in an extended swollen state. At $\tau >\tau_{\mathrm{collapse}}$ (above the transition lines) the PE brush transits to collapsed state (two-phase regions are not shown). At $\tau \gg \tau_{\mathrm{collapse}}$ the brush acquires thickness $D_{\mathrm{collapsed}}$ $≃{\left(na^{3}/h\tau \right)}^{1/2}$, and the thermal blobs with constant size $\xi_{T}≃a/\tau$ (shown in grey color in Figure 5) are densely packed in the uniform collapsed layer.

At loose grafting of the ligands onto the cylindrical surface, collapsed polymer chains organize in the so-called octopus [61] micelles comprising several tethered chains, or stay as uni-molecular globules [62]. They could exhibit also more complex helicoidal structures [36] and mixed morphologies [63] at nonuniform grafting of polymer chains.

### 2.5. Effect of the Backbone Charge Density

The presence of ionizable sites on the backbone of cylindrical PE brush, e.g., in polymer-decorated cellulose nanocrystals (CNCs), with surface ionizable groups not involved in ligand grafting or alternative surface modifications) could shift the boundaries between different regimes. For long polyions, the presence of similar charges on the backbone with linear density α and extra counterions in the brush (αh per polyion) does not significantly affect the power law dependences for the brush thickness *D* and the boundaries between different regimes if βn≫αh. It reduces to substitution β→ (β+αh/n) in all equations incorporating β.

If αhe<0 (i.e., the backbone bears αh charges per ligand, opposite in sign to the charges on polyion), tethered polyions could partition in two populations, similar to what happens in a planar PE brush tethered to an oppositely charged substrate [64]. To compensate nanorod surface charge, fraction γ=αh/βn of polyions could relocate from the brush forming an adsorbed layer around the backbone, while the rest will remain in the stretched conformations with, however, decreased grafting density. That is, the distance between neighboring chains in the remaining (extended) part of the PE brush would increase as h/(1−γ) to substitute *h* in all equations. While the number of counterions βn per polyion in the extended population is not changed in this case, the decreased grafting density of ligands, (1−γ)/h, could shift the osmotic PE brush closer to the O-C boundary (see the diagram of states in Figure 2), and lead to the regime change, transforming osmotic (O) into the charged brush (C) and further to isolated polyions (IS).

An alternative scenario is delegation of ∆n=αh/β=nγ monomer units by each polyion to compensate for the backbone charge while remaining polyions with a decreased degree of polymerization n−∆n=n(1−γ) and distance *h* between neighboring chains forms osmotic PE brush with thickness $D≃a\beta^{1/2}n\left(1-\gamma \right)$.

**Figure 6:**
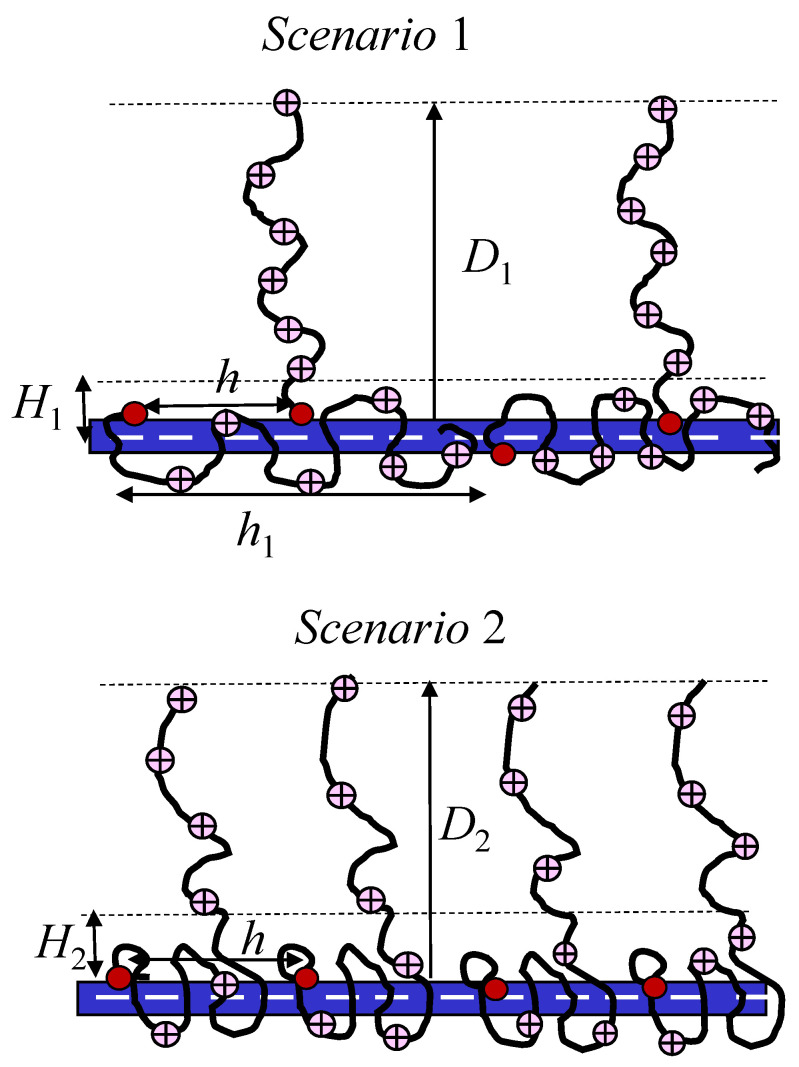
Schematics of two scenarios for compensation of rod charge by tethered polyions. Red circles mark grafting points of tethered polyions.

Compare the free energies *F* per tethered polyion in the two systems: (1) osmotic PE brush with distance $h_{1}=$h/(1−γ) between chains with DP *n*, thickness $D_{1}≃a\beta^{1/2}n$, and fraction γ=αh/βn≤1 of adsorbed polyions, and (2) osmotic PE brush with DP n(1−γ), distance *h* between grafts, thickness $D_{2}≃a\beta^{1/2}n\left(1-\gamma \right),$ and number αh/β=nγ of monomer units in the adsorbed layer per polyion (see schematics in Figure 6).

In the first scenario, the average concentration $c_{1}$ of counterions in the the remaining PE brush is given by $c_{1}≃\beta n/h_{1}D_{1}^{2}≃\left(1-\gamma \right)a/hn,$ and transfer of nβ counterions from each adsorbing polyion in the solution with Debye screening length $r_{D}\gg D_{1}$ leads to the translational entropy gain

(11) $$\frac{F_{1,ion}}{k_{B}T}≃\beta n\ln\frac{a(1-\gamma )}{hnc_{s}}\;$$

The same amount of counterions is transferred from the vicinity of the charged backbone in the solution. The remaining brush polyions are stretched normally to the rod surface and have the elastic free energy per chain

$$\frac{F_{1,normal}}{k_{B}T}≃\frac{D_{1}^{2}}{a^{2}n}≃\beta n$$

In order to compensate for the backbone charge, the adsorbed polyions elongate to acquire the end-to-end distance $h_{1}=a\beta n/\alpha$, and the elastic free energy $F_{1,lateral}/k_{B}T≃$ $h_{1}^{2}/a^{2}n≃n{\left(\beta /\alpha \right)}^{2}$. The adsorbed polyion is envisioned as a string of $an/h_{1\;}$ elastic blobs with size $H_{1}≃a\alpha /\beta$ each, and the electrostatic attraction energy $W_{1}/k_{B}T≃l_{B}a\beta n\ln\left(H_{1}/r_{D}\right)$. Notably, $W_{1}$ specifies the difference between the electrostatic energy of the adsorbed layer of polyions (i.e., a capacitor with outer plate at distance $H_{1}$ from the backbone) and the reference state of an uncompensated charged cylinder (capacitor with outer plate at a distance $r_{D}$ from the backbone). Then the free energy $F_{1}$ is given by

$$\frac{F_{1}}{k_{B}T}≃\left(1-\gamma \right)\left(\frac{F_{1,ion}}{k_{B}T}+\frac{F_{1,normal}}{k_{B}T}\right)+\gamma \left(\frac{F_{1,lateral}}{k_{B}T}+\frac{W_{1}}{k_{B}T}\;\right)≃\;\;$$

(12) $$\left(1-\gamma \right)\beta n\left(\ln\frac{(1-\gamma )a}{hnc_{s}}+1\;\right)+\left(\frac{h\beta }{a^{2}\alpha }+\alpha^{2}l_{B}h\ln\left(\frac{H_{1}}{r_{D}}\right)\right)\;\;\;$$

Similar arguments in scenario 2,

$$\frac{F_{2}}{k_{B}T}≃\left(\frac{F_{2,ion}}{k_{B}T}+\frac{F_{2,normal}}{k_{B}T}\right)+\left(\frac{F_{2,lateral}}{k_{B}T}+\frac{W_{2}}{k_{B}T}\;\right)$$

with concentration $c_{2}≃\beta n\left(1-\gamma \right)/hD_{2}^{2}≃a/\left[hn\left(1-\gamma \right)\right]$ in the normally stretched part of the PE brush, and adsorbed layer envisioned as an asymmetric PEC [65] with thickness $H_{2}≃\xi_{e}\left(\beta \right)≃a\beta^{-2/3}$, total number $h/\xi_{e\;}$ of the electrostatic blobs with total elastic free energy $F_{2,lateal}/k_{B}T≃$$h^{2}/a^{2}∆n≃h^{2}/\left(a^{2}n\gamma \right)≃h\beta /\left(a^{2}\alpha \right)$, and electrostatic energy $W_{2}=$$l_{B}\alpha^{2}h\ln\left(H_{2}/r_{D}\right)$ per tethered polyion give

(13) $$\frac{F_{2}}{k_{B}T}≃\beta n\left(1-\gamma \right)\left(\ln\frac{a}{nh\left(1-\gamma \right)c_{s}}+1\right)+\left(\frac{h\beta }{a^{2}\alpha }+l_{B}\alpha^{2}h\ln\left(\frac{H_{2}}{r_{D}}\right)\right)$$

Notably, in both $F_{1}$ and $F_{2}$, the second term in the first round brackets is due to elastic stretching of polyions in PE brush. The difference between the free energies in the two considered systems is:

(14) $$\frac{∆F}{k_{B}T}=\frac{F_{2}-F_{1}}{k_{B}T}≃-2\left(1-\gamma \right)\beta n\ln\left(1-\gamma \right)+l_{B}\alpha^{2}h\ln\left(\frac{H_{2}}{H_{1}}\right)$$

The first term in Equation (14) is always positive, indicating thermodynamic preference of state (1), i.e., partitioning of polyions in two populations. The sign of the second term in Equation (14) is governed by the conformations of adsorbed polyions. However, the absolute value of the second term is smaller than of the first one if αa≪1, i.e., in the considered here range of α.

Therefore, disproportionation of polyions in two populations in case of overcompensation of the rod charge by the tethered polyions is always thermodynamically favorable.

Importantly, the remaining stretched polyions could ensure the stability of the polymer-decorated dispersion if γ<1.

### 2.6. Comparison to Experiments

The transition from a swollen to a collapsed state of cylindrical polymer brush is linked to the loss of thermodynamic stability of dispersed polymer-decorated nanorods. The onset of collapse (specified by the reduced temperature, $\tau_{\mathrm{collapse}}$) depends on the salt concentration $c_{s}$, DP *n* and ionization degree β of the tethered chains, and their grafting density $h^{-1}$. By varying molecular parameters of the tethered chains (e.g., degree of ionization β in case of pH-responsive ligands) or temperature *T* (e.g., changing τ for thermo-responsive ligands) one could regulate coagulation and onset of gelation in dispersions of polymer-decorated nanorods. Many experimental studies focus on polymer-decorated CNCs (see, e.g., review [66] and references therein). Unfortunately, in spite of the enormous amount of publications on decorated CNC dispersions, we did not find systematic data on the polymer layer thickness *D* as a function of the molecular parameters of tethered ligands, and thereby comparison between the predictions of our model and the selected experimental data is semi-quantitative.

The thermo-responsiveness of CNCs decorated by grafted non-ionic poly(N-isopropylacrylamide) (PNIPAAm) chains were investigated in detail in ref. [67]. Undecorated CNCs exhibited negative zeta potential at neutral pH that was decreasing in absolute value upon ligands grafting, indicating “shielding effect” of polymer chains. Two water dispersions of CNCs with length L≃300 nm and width ≃10 nm decorated by PNIPAAm chains with almost equal DP n≃350, but different (theoretically estimated) grafting densities σ=0.07 (PNIPAAm-CNC-1) and 0.02 (PNIPAAm-CNC-2) chains/nm${}^{2}$ were examined. At temperature T< 34 ${}^{\circ }$C, the tethered PNIPAAm chains were extended with (experimentally estimated from DLS) thickness H≈15 nm (PNIPAAm-CNC-1) and 9 nm (PNIPAAm-CNC-2), comparable to the diameter of CNC and both PNIPAAm grafted CNC suspensions were homogenous. Low absolute values of zeta potential prompted the stabilization of polymer-decorated CNCs in this range of temperatures were steric (not electrostatic) due to repulsions between tethered macromolecules. Upon heating above 34 ${}^{\circ }$C, both of the PNIPAAm grafted CNC suspensions experienced a sharp transition to an unstable gel. The transition temperature T= 34 ${}^{\circ }$C (estimated from the sharp increase in the dynamic storage modulus) was higher than the LCST ≈30.5 ${}^{\circ }$C of the free PNIPAAm. Above the transition temperature, T> 34 ${}^{\circ }$C, PNIPAAm chains formed thin collapsed layers, and polymer-induced hydrophobic inter-particle attractions resulted in the coagulation of grafted CNC particles.

As follows from the comparison between sizes of grafted PNIPAAm chains and underlying CNC with diameter $D_{\mathrm{CNC}}≃10$ nm, the geometry of the polymer layer could be considered intermediate between planar and cylindrical. Small absolute values of zeta potential prompt that the effect of residual charges on the CNC surface can be, in the first approximation, neglected. We, therefore, use the prediction for the collapse transition of PE brush in a quasi-neutral (QN) regime (Equation (8)), which is also applicable to neutral cylindrical brushes. If one associates LCST ≈30.5${}^{\circ }$C with θ-temperature of PNIPAAm solution, then the experimental transition temperature $T_{\mathrm{collapse}}=34$${}^{\circ }$C corresponds to $\tau_{\mathrm{collapse}}^{\exp}$$=\left(T_{\mathrm{collapse}}-LCST\right)/\left(273+T_{\mathrm{collapse}}\right)\approx \left(34-30.5\right)/307≃10^{-2}$. The reported grafting densities σ=0.07 and 0.02 chains/nm${}^{2}$ correspond to linear distances h=${\left(\pi D_{\mathrm{CNC}\;}\sigma \right)}^{-1}$ ≈ 0.45 nm and 1.6 nm between neighboring chains, respectively. Taking into account PNIPAAm cross-section 0.2 nm${}^{2}$ and implementing size of monomer unit as $a=\sqrt{0.2{\mathrm{nm}}^{2}}\approx 0.45$ nm, one estimates the theoretical transition point (Equation (8)) as $\tau_{\mathrm{collapse}}^{\left(\mathrm{QN}\right)}≃{\left(a/nh\right)}^{1/3}≃0.14$ (PNIPAAm-CNC-1) and 0.09 (PNIPAAm-CNC-2). An alternative estimate using the theoretical prediction for the collapse of a quasi-neutral planar brush [41]: $\tau_{\mathrm{collapse}}≃a\sigma^{1/2}≃$ 0.12 and 0.06, respectively. Both types of theoretical estimates are of the same order of magnitude, consistent with the intermediate geometry of the PNIPAAm brush. The correspondence between $\tau_{\mathrm{collapse}}^{\exp}$ and $\tau_{\mathrm{collapse}}^{\left(\mathrm{QN}\right)}$ also seems reasonable, with smaller value of $\tau_{\mathrm{collapse}}^{\exp}$ pointing at possible overestimation of the grafting densities of PNIPAAm chains. The numerical discrepancy between theoretical ($≃10^{-1}$) and experimental ($≃10^{-2}$) values of the reduced deviations of $T_{\mathrm{collapse}}$ from LCST could also arise due to the numerical coefficients omitted in the scaling models.

Variations in LCST of polymer-decorated CNCs allow for tunable loss of dispersion thermal stability [68,69]. In a series of Poly(PEGMA)-g-CNCs, the monomer in the side chain was composed of a methacrylate group that forms the polymer backbone after polymerization, and ethylene glycol side chains with varying length, and thereby the synthesized grafts constitute flexible molecular brushes with variable degree of polymerization. Poly(PEGMA)-g-CNCs exhibit thermo-responsive behavior in aqueous solution with the LCST in the range of 34–66 ${}^{\circ }$C, increasing with a ratio of OEGMA in the comonomer feed. Above LCST rod-like nanostructures aggregated into spherical nanoparticles with loose or dense packing of CNC nanorods. This thermally-induced aggregation was fully reversible upon cooling. Remarkably, the experimentally measured LSCT for Poly(PEGMA)-g-CNCs dispersions were lower than the LCST for free Poly(PEGMA) chains under similar conditions [68]. That is, the solution of Poly(PEGMA) chains was more stable (with LCST higher by ∆T≃2–4 ${}^{\circ }$C) than Poly(PEGMA)-g-CNCs dispersions. According to the theory [40], tethered polymers in the brush give rise to larger polymer volume fraction than individual tethered coils at loose grafting. Therefore, CNC stabilized by polymer layers are expected to lose stability at larger temperatures *T* than pristine CNCs provided that: (i) tethered chains form a brush, and (ii) surface CNC charge is negligible. However, the observed opposite trend points to the possible effect of CNC surface charges. In the presence of tethered polymer, surface CNC charge might be shielded stronger by the dense collapsed brush compared to a swollen one, i.e., the brush collapse might effectively decrease CNC surface charge and shift dispersion LCST to lower temperatures, overruling the predicted increase in LCST due to dense grafting of the ligands.

The pH-responsiveness of CNCs has been introduced by grafted ionizable poly(acrylic acid) (PAA) [33] and poly(4-vinylpyridine) (P4VP) [70] chains. PAA chains were obtained by acid hydrolysis of preliminary synthesized densely grafted (up to 0.3 chains/nm${}^{2}$) P*t*BA brushes with controllable and variable in a wide range DPs. The former gave rise to a cylindrical brush around CNC with a thickness considerably exceeding the CNC diameter ≃7 nm. Although variations in pH affect the ionization of PAA ligands (value of β), no systematic data on the pH-triggered variations in the brush thickness were reported that could be compared to the theory.

The pH responsiveness has been clearly demonstrated for hybrids with P4VP chains grafted from CNC surface hydroxyl groups via ceric-ion-initiated polymerization in water [70]. P4VP is a weak cationic polyelectrolyte with a $pK_{a}\approx 5$. At pH >5, P4VP becomes hydrophobic due to deprotonation of the pyridyl groups. The turbidity and electrophoretic mobility experiments demonstrated the complex behavior of P4VP-g-CNCs aqueous suspensions with flocculation and sedimentation above pH 5. The negative surface charge of P4VP-g-CNCs above pH 5 was attributed to the CNCs’ anionic sulfate ester groups, which are present after the grafting reaction with estimated charge density $\sigma_{sulf}=0.34$ e/nm${}^{-2}$, giving the Gouy–Chapman length $\lambda ={\left(2\pi l_{B}\sigma_{sulf}\right)}^{-1}\approx 0.7$ nm ≃a. Although DP *n* of tethered polymer was not specified, the estimated from elemental analysis total number of polymer ionizable groups per CNC, $N_{PVP}$, was almost three times larger than the total number $N_{sulf}$ of surface sulphate ester groups, $N_{PVP}/N_{sulf}≃3$. Comparable in absolute values electrophoretic mobilities of pristine CNCs and P4VP-g-CNCs at pH ≃4 (i.e., when the P4VP brush is cationic with degree of ionization β>0.5) indicate that even in the presence of pH-insensitive CNC surface charges, aqueous P4VP-g-CNCs dispersion is stabilized at pH ≤ 5 by the charged P4VP polyions.

Increases in pH decrease fraction β of charged PVP groups in the tethered chains, and increase the ratio $N_{sulf}/\beta N_{PVP}$. According to the scaling model discussed above, fraction $N_{sulf}/\beta N_{PVP}$ of monomers in the tethered P4VP chains should compensate for surface charge with the remaining stretched polyions separated by average distance $≃h/(1-N_{sulf}/\beta N_{PVP})$ along the CNC core. At $\beta =\beta_{*}=N_{sulf}/N_{PVP}\approx 0.3$ (i.e., at pH >5) all tethered polyions transfer to the CNC surface and form adsorbed layer to compensate surface charge, and a further increase in pH (i.e., the decrease in $\beta <\beta_{*\;}$) leads to the surface charge undercompensation. Presuming that at pH ≃4 and fixed experimental temperature $T_{0}$ (i.e., $\tau_{0}=\left(\theta -T_{0}\right)/T_{0}=const$), the tethered polyions in the extended part of the PE brush are found in the osmotic regime (low salt), so that $\tau_{\mathrm{collapse}\;}($pH $4)=\tau_{\mathrm{collapse}\;}^{(O)}≃\beta^{1/2}>\tau_{0}$, an increase in pH >4 leads to the decrease in β, and the corresponding decrease in $\tau_{\mathrm{collapse}\;}≃\beta^{1/2}$. If $\tau_{\mathrm{collapse}\;}\left(\beta_{*}\right)\equiv \tau_{*\;}$ (with $\beta_{*\;}$ corresponding to neutralization of surface charge) exceeds $\tau_{0\;}$, $\tau_{0}<\tau_{*}$, then all polyions remain under theta-conditions in the swollen adsorbed layer at $\beta <\beta_{*}$, and only when $\tau_{\mathrm{collapse}\;}\left(\beta \right)$ reduces down to $\tau_{0\;}$, temperature $T_{0}$ becomes the transition temperature $T_{\mathrm{collapse}\;}$, the adsorbed polymer layer collapses, and aqueous P4VP-g-CNCs dispersion loses stability. If $\tau_{0}>\tau_{*}$, then collapse is linked to relocation of polyions to the CNC surface accompanied by loss or dispersion stability. Both scenarios are consistent with the observed experimental trend: loss of stability of polymer-decorated CNCs below pH 5, although we could not distinguish between $\tau_{0}$ and $\tau_{*}$ from the experimental data. Clearly, more systematic experiments on well characterized PE cylindrical brushes are necessary to perform comprehensive comparison between the theoretical predictions and the experimental data.

## 3. Conclusions

In this study we focused on the effects of solvent strength and ionic charge of the backbone on the conformational properties of cylindrical PE brushes with the main emphasis on the onset of the transition from swollen (extended) to collapsed states of the brush.

Inferior solubility of the tethered polyions (via solvent quality, variations in pH and ionic strength, etc.) led to collapse of the PE brush, concomitant loss of its protective (stabilizing) properties, and onset of coagulation/gelation of polymer-decorated dispersions. The transition of a relatively weakly charged PE brush to the collapsed state from osmotic and salt-dominated regimes was promoted by the decrease in degree of polyion ionization β, and increase in the salt concentration $c_{s}$, while collapse from quasi-neutral state of PE brush was governed mostly by DP *n* and grafting density $h^{-1\;}$ of the tethered polyions. The developed theoretical model could be applied to rationalize the experimental data on dispersions of polymer-decorated cellulose nanocrystals (CNCs) with a focus on tunable dispersion stabilization/destabilization.

The scaling model of PE cylindrical brush could be also used to compare the equilibrium swelling ratios of hairy gels with ionized and neutral side chains with similar architectures of the strands, i.e., with same DP *M* of the backbone and DP *n* of the side chains, separated by spacers with DP *m* (n/m≪1). In the hollow mesh regime, the swelling coefficient $Q_{gel}$ is specified by the average end-to-end distance $R_{backbone}$ of the backbone, and the total DP of the strand, N=M(1+n/m) as $Q_{gel}=R_{backbone}^{3}/a^{3}N$. At fixed *N*, ionization of the bottlebrush side chains in salt-free solution (osmotic regime O with $\beta >{\left(nm\right)}^{-2/3}$) led to stretching of the backbone up to its contour length, h≃ma, L≃aM, and $R_{backbone}\left(\beta \right)≃L$. In contrast, neutral side chains with β=0 exhibited self-avoiding statistics on length scales larger than the brush thickness *D*, and hairy gels with such strands had $R_{backbone}\left(\beta =0\right)∼aM^{3/5}$, supplemented with weakly increasing dependence on *n* (specified by the degree of local stretching of spacers) [26]. Therefore, ratio of the swelling coefficients of the osmotic and neutral hairy gels in hollow mesh regime, $Q_{gel}\left(\beta \right)/Q_{gel}\left(\beta =0\right)∼M^{3}/M^{9/5}∼M^{6/5\;}$, strongly increased with DP *M* of the backbone and exhibited weakly decreasing dependence on DP *n* of the side chains. Specifics of the charged hairy gel behavior in the filled mesh regime, and the effect of ionic strength will be examined in our following publications.

## Figures and Tables

**Table 1:** Equilibrium parameters of cylindrical PE brush in various regimes of diagram of states in Figure 2.

Regimes	D/a	c(r)
*charged* (C)	$\beta n^{3/2}{\left(a/h\right)}^{1/2}$	−
*osmotic* (O)	$\beta^{1/2}n$	$\beta^{-1/2}\left(a^{2}/hr\right)$
*quasi-neutral* (QN)	$n^{2/3}{\left(a/h\right)}^{1/3}$	${\left(a^{2}/hr\right)}^{1/2}$
*isolated stretched* (IS)	$\beta^{2/3}n$	$\beta^{-2/3}{\left(a/r\right)}^{2}$
*Gaussian* (G)	$n^{1/2}$	a/r

**Table 2:** *Power law dependences for PE brush thickness D, size of elastic blob* ξ(r), *and polymer density profile* c(r) *in salt-added solution* $l_{B}/a≃1$*,* α=0, ${\left(nh/a\right)}^{-2/3}<\beta <{\left(nh/a\right)}^{-1/2}$.

	Regime O	Regime S	Regime QN
$c_{s}$	$0<c_{s}<a{\left(nh\right)}^{-1}$	$a{\left(nh\right)}^{-1}<c_{s}<a{\left(\beta^{2/3}nh\right)}^{-1}$	$a{\left(\beta^{2/3}nh\right)}^{-1}<c_{s}<1$
D/an	$\beta^{1/2}$	${\left(a^{2}\beta^{2}/c_{s}nh\right)}^{-1/4}$	${\left(hn/a\right)}^{-1/3}$
ξ(r)/a	$\beta^{-1/2}\;$	${\left(c_{s}/\beta^{2}\right)}^{1/3}{\left(a^{2}/rh\right)}^{2/3}$	${\left(a^{2}/rh\right)}^{1/2}$
c(r)	$\beta^{-1/2}a^{2}/rh$	${\left(a/nh\right)}^{-2/9}{\left(\beta^{2}/c_{s}\right)}^{1/3}\;$	${\left(rh/a^{2}\right)}^{3/7}{\left(c_{s}/\beta^{2}\right)}^{1/7}$

## Data Availability

Not applicable.
